# An Overview of Australian Podiatry Research: A Bibliometric Review

**DOI:** 10.1002/jfa2.70113

**Published:** 2026-02-14

**Authors:** Peta Tehan, Ameer Nor Azhar, Helen Banwell, Shan Bergin, James Charles, Fiona Hawke, Malia Ho, Sheree Hurn, Michelle Kaminski, Polly Lim, Saraid Martin, Hylton B. Menz, John Osborne, Benjamin Peterson, Dean Samaras, Cylie Williams, Matthew R. Carroll

**Affiliations:** ^1^ School of Clinical Sciences, Faculty of Medicine, Nursing and Health Sciences Monash University Clayton Victoria Australia; ^2^ School of Health Sciences, College of Medicine, Health and Welleing University of Newcastle Newcastle New South Wales Australia; ^3^ Discipline of Podiatry, School of Allied Health, Human Services and Sport La Trobe University Melbourne Victoria Australia; ^4^ Adelaide University, Allied Health and Human Performance Adelaide South Australia Australia; ^5^ First People's Unit, School of Health Sciences and Social Work Griffith University Gold Coast QueensLand Australia; ^6^ School of Primary and Allied Health Care Monash University Melbourne Victoria Australia; ^7^ School of Clinical Sciences, Faculty of Health Queensland Unviesrity of Technology Brisbane QueensLand Australia; ^8^ Department of Podiatry Monash Health Melbourne Victoria Australia; ^9^ Wardliparingga Aboriginal Health Equity SAHMRI Adelaide South Australia Australia; ^10^ Department of Podiatry, School of Health, Medical and Applied Sciences CQUniversity Rockhampton QLD Australia; ^11^ Melbourne Podiatric Surgery Melbourne Victoria Australia; ^12^ Department of Podiatry, School of Allied Health Auckland University of Technology Auckland New Zealand

## Abstract

**Background:**

Podiatrists are the primary health professionals associated with assessment, diagnosis and management of lower limb problems. Research is critical in informing evidence‐based practice. As part of a national research priorities project, this bibliometric review aimed to map all Australian podiatry‐relevant research from 1970 to 2024 and explore volume over time, authors, institutions, level of evidence, funding sources and categories of research.

**Methods:**

Podiatry‐relevant research was categorised into 10 streams: dermatology, diabetes‐related foot disease, gerontology, musculoskeletal and sports, paediatrics, rheumatology, surgery, workforce and education, First Nations foot health and neurological and vascular disease. A systematic search of the literature was conducted in each stream up until December 2024. Meta‐data from Scopus were analysed in Biblioshiny, where publications volume, authors, institutions, journals and collaborations were described. Each publication was also categorised for level of evidence using the National Health and Medical Research Council criteria, research type using the United Kingdom Clinical Research Collaboration Health Research Classification System and funding source using Higher Education Research Data Collection specifications.

**Results:**

A total of 1641 publications were included across all research streams. Steady increases in publication volume occurred over the past 20 years, with diabetes‐related foot disease yielding the highest volume (*n* = 335), followed by musculoskeletal (*n* = 308) and paediatrics (*n* = 280). Musculoskeletal and sports research demonstrated the highest proportion of level I evidence (22%), whereas most streams were dominated by level IV evidence. The majority of research across all streams received no funding support, ranging from 32% unfunded in First Nations foot health research to 87% in surgical research. Rheumatology achieved the highest proportion of competitive funding (47% Category 1). The most frequent research categories were aetiology, detection and screening and evaluation of treatments. The Journal of Foot and Ankle Research was the most frequent publication source, with 140 (8%) of total publications.

**Conclusion:**

Australian podiatry‐relevant research has grown substantially, particularly over the past 2 decades. However, significant disparities exist in volume, evidence quality and funding across different streams, with most research conducted without external funding support, highlighting the need for strategic investment to enhance evidence generation in key areas of podiatry practice.

## 1 Background

Podiatrists are the primary health professionals involved in the assessment, diagnosis and management of lower limb problems. The scope of podiatry is broad, encompassing the clinical care of conditions related to the neurological, vascular, rheumatological, endocrine and musculoskeletal systems. It also includes specialised care across diverse populations such as paediatrics and gerontology, foot and ankle surgery and First Nations foot health. This breadth of practice reflects the complex and multifaceted nature of lower limb health and highlights the diverse clinical expertise required to deliver contemporary podiatry care.

Podiatry in Australia is a growing and evolving profession, with 6285 registered podiatrists currently practising across a diverse range of settings, including public and private healthcare, aged care, education and research [1, 2]. This represents a substantial increase from 2500 practitioners in 2002, reflecting more than a 100% growth over 2 decades and highlights the growing need for skilled lower limb healthcare and the expanding scope of practice for the podiatry workforce [3].

Research is fundamental to evidence‐based practice and professional advancement in podiatry [4, 5]. However, the current landscape of Australian podiatry research has not been systematically mapped, making it difficult to identify research strengths, gaps and priorities across the diverse domains of podiatric practice. Understanding research trends, funding patterns and evidence quality is essential for strategic research planning and resource allocation to advance the profession and ultimately improve patient outcomes.

Bibliometric reviews are a contemporary research method which enable researchers to map large volumes of research to identify: trends, volume over time, author relationships, international collaborations and impact of outputs over time. Bibliometric reviews can provide valuable insight, particularly in relation to a research discipline or topic area [6]. As part of a broader strategy to identify national research priorities, this bibliometric review aimed to map existing podiatry‐related research conducted by Australian researchers in Australian cohorts. Results from this review will inform a nationwide Delphi exercise, which aims to determine national research priorities within the profession.

## 2 Methods

This study was a bibliometric analysis between January 1970 and December 2024. The Scopus database was used due to its large abstract and citation database, the ability to search by publication, author or affiliation and its congruence with Biblioshiny application (R version 3.6.1, Bibliometrix package version 2.2.1; University of Naples Federico II, Naples, Italy, 2016). Scopus also offers a broader range of journals compared to PubMed and Web of Science [7].

### 2.1 Search Strategy

Separate search strategies were derived for the following themes: dermatology, diabetes‐related foot disease, gerontology, musculoskeletal and sports, paediatrics, rheumatology, surgery, workforce and education, First Nations foot health, and neurological and vascular disease. Search strategies are described in Supporting Information S1: Table S1. Similarly, Preferred Reporting Items for Systematic reviews and Meta‐Analyses (PRISMA) flow charts for each stream are also presented in Supporting Information S1.

### 2.2 Study Selection

Results of the Scopus search were downloaded into Covidence (Veritas Health Innovation, Melbourne, Australia) online review software. Titles, abstracts and full text articles were then independently screened by two researchers in each stream, with disagreements resolved by discussion or a third researcher. Eligible publications were original articles or systematic reviews published in English, completed at an Australian education or healthcare institution, in an Australian cohort of participants, where at least one author had an Australian affiliation. For systematic reviews, the first or last author was required to have had an Australian affiliation. Eligible research was deemed to be clinically relevant if it demonstrated applicability to podiatry practice, specifically, if its findings could inform clinical decision‐making or patient care, workforce planning and continuing education. Laboratory‐based studies or preclinical studies were not included as they were not deemed to be directly relevant to podiatry clinical practice. Due to the breadth of research in musculoskeletal and sports, this topic was narrowed to the foot and ankle only. The neurovascular stream included research focused specifically on assessment and/or management of neurological or vascular conditions affecting the lower limb. Guidelines, consensus documents, case studies, research letters, editorials, commentaries and conference abstracts were not included.

### 2.3 Data Extraction

Publication metadata for all included studies were retrieved from Scopus and analysed using Biblioshiny (R version 3.6.1, Bibliometrix package version 2.2.1; University of Naples Federico II, Naples, Italy, 2016) for data analysis. The following bibliometric measures were obtained for each study: publication year, journal title, citation count (as recorded in the Scopus database [Elsevier, Amsterdam, Netherlands]), author details, number of authors per paper, and institutional and geographic affiliations for all authors.

### 2.4 Data Synthesis

To investigate the level of evidence using the National Health and Medical Research Council criteria, that was being generated across the different streams, this was manually assigned to each study by a single researcher in each stream [8]. Research focus was similarly categorised according to the United Kingdom Clinical Research Commission (UKCRC) Health Research Classification System [9], which has been described previously [10]. Both the NHMRC and UKCRC classifications are outlined in Supporting Information S1: Tables S1 and S2. Funding categories were also extracted from each study where reported, according to the Australian Government Higher Education Research Data Collection (HERDC) specifications, which has been described previously [10]. The accuracy of manual coding was checked with a second researcher (PT) checking 10% of the coding, while blinded to the original coding.

## 3 Results

**FIGURE 1:**
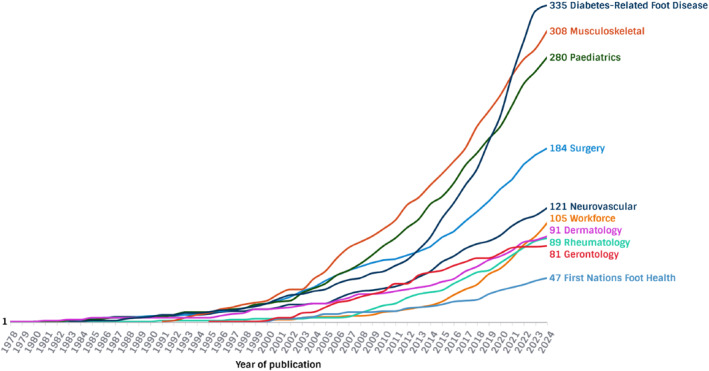
Number of publications over time.

**FIGURE 2:**
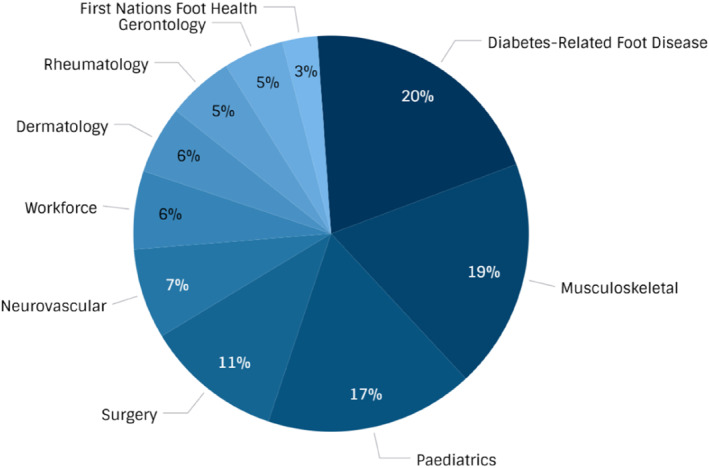
Research in each stream.

A total of 1641 publications were included (Figure 1). A total of 164 publications (10%) were included in more than one stream. Combined publication characteristics are presented in Table 1 and breakdown in each stream is presented in Table 2 and Figure 2. Diabetes‐related foot disease yielded the highest number of total publications (*n* = 335, 20%), followed by musculoskeletal and sports (*n* = 308, 19%) and paediatrics (*n* = 280, 17%). The smallest number of publications were in gerontology (*n* = 81, 5%) and First Nations foot health (*n* = 47, 3%). The highest number of systematic reviews was reported in musculoskeletal and sports (*n* = 56; 18%). Gerontology had the highest number of mean citations per article (*n* = 62) whereas the workforce and education stream had the smallest number of mean citations per article (*n* = 9). The most frequently targeted journals by Australian authors were *The Journal of Foot and Ankle Research* (*n* = 140; 85%), *Gait and Posture* (*n* = 67; 4%) and *The Journal of the American Podiatric Medical Association* (*n* = 48; 3%). *The Journal of Foot and Ankle Research* was in the top three journals across nine of the 10 streams of research. Most globally cited articles is outlined in Table 3.

**TABLE 1:** Combined publication characteristics.

	Diabetes‐related foot disease	Musculoskeletal	Paediatrics	Surgery	Neurovascular	Workforce/education	Dermatology	Rheumatology	Gerontology	First Nations foot health
Publication range	1983–2024	1991–2024	1980–2024	1980–2024	1981–2024	1998–2024	1978–2024	1983–2024	1995–2024	1998–2024
Total number of publications	335	308	280	184	121	105	91	89	81	47
Original publications, *n* (%)	290 (87)	252 (82)	254 (91)	165 (90)	102 (84)	99 (94)	83 (91)	80 (90)	76 (94)	41 (87)
Systematic reviews, *n* (%)	45 (13)	56 (18)	26 (9)	19 (10)	19 (16)	6 (6)	8 (9)	9 (10)	5 (6)	6 (13)
Mean years from publication	8.97	11.6	11.10	12.3	12.3	6.72	12.9	9.38	13.3	9.89
Mean citations per year per publication	18.1	44.5	29.6	25.0	29.8	9.30	27.27	27.40	62.0	17.9
Total citations	6076	13,697	8331	4603	3608	975	2482	2438	5024	843
Total authors	1043	819	793	659	443	338	437	200	177	173
Mean coauthors per publication	3	4.6	5.1	4.5	5.1	4.5	5.43	5.6	4.3	5.22
International coauthorships (%)	29.6	19.2	26.8	31.5	24.8	13.3	26.4	30.3	17.3	14.9
Single‐authored publications	3	7	5	4	0	6	4	0	2	0

**TABLE 2:** Characteristics of publications stratified by research streams.

	Diabetes‐related foot disease	Musculoskeletal	Paediatrics	Surgery	Neurovascular	Workforce	Dermatology	Rheumatology	Gerontology	First Nations foot health
Top three journals publishing the most Australian research, *n* (%)	*Journal of Foot and Ankle Research,* 56 (17) *Diabetic Medicine,* 23 (7) *International Wound Journal,* 15, (4) and *Journal of Diabetes and its Complications,* 15 (4)	*British Journal of Sports Medicine,* 25 (8) *Journal of Science and Medicine in Sport*, 23 (7) *Journal of Foot and Ankle Research*, 22 (7)	*Gait and Posture,* 35 (12) *Journal of Foot and Ankle Research*, 20 (7) *Developmental Medicine and Child Neurology*, 13 (5)	*Foot and Ankle International*, 30 (16) *Journal of Foot and Ankle Surgery*, 12 (6) *Foot and Ankle Surgery*, 11 (6) *Journal of Foot and Ankle Research*, 11 (6)	*Journal of Vascular Surgery*, 8 (7) *Journal of Foot and Ankle Research*, 7 (6) *Diabetic Medicine*, 6 (5)	*Journal of Foot and Ankle Research*, 34 (32) *Australian Health Review*, 7 (7) *Journal of the American Podiatric Medical Association*, 7 (7)	*Australasian Journal of Dermatology*, 12 (13) *Internal Medicine Journal,* 5 (6) *International Wound Journal*, 5 (6) *Journal of Foot and Ankle Research*, 5 (6) *Journal of the American Medical Association*, 5 (6)	*Arthritis Care and Research*, 16 (18) *Osteoarthritis and Cartilage*, 13 (15) *Journal of Foot and Ankle Research*, 7 (8)	*Gait and Posture*, 12 (15) *Journal of the American Podiatric Medical Association*, 7 (9) *Gerontology*, 5 (6)	*Journal of Foot and Ankle Research*, 9 (19) *Australian Journal of Primary Health*, 3 (6) *Australian Journal of Rural Health*, 3 (6)
Top three most frequent authors, *n* (%)	Lazzarini P.A., 40 (12) Chuter V.H., 28 (8) Golledge J.A., 24 (7)	Menz H.B., 47 (15) Larndorf K.B., 30 (10) Vicenzino B., 25 (8)	Williams C.M., 39 (14) Burns J., 37 (13) Steel J.R., 14 (5)	Saxby T.S., 13 (7) Dearden P.M.C., 7 (4) Platt S.R., 7 (4)	Chuter V.H., 32 (26) Tehan P.E., 15 (12) Golledge J., 12 (10)	Williams C.M., 21 (20) Menz H.B., 13 (12) Causby R.S., 11 (10)	Kaminski M.R., 4 (4) Frescos N., 3 (3) Jones S., 3 (3) Landorf K.B., 3 (3) McMahon L.P., 3 (3) Nixon R. 3, (3) Raspovic A., 3 (3)	Menz H.B., 35 (39) Munteanu S.E., 28 (31) Hinman R.S., 26 (29)	Menz H.B., 50, (62) Lord S., 23 (28) Landorf K.B., 11 (14)	Chuter V.H., 7 (15) West M., 7 (15) Charles J., 5 (11) Hawke F., 5 (11) Sadler S., 5 (11)
Top three most frequent institutional affiliations, n	Queensland University of Technology, 116 University of Newcastle, 93 James Cook University, 84	La Trobe University, 210 University of Sydney, 181 University of Queensland, 79	University of Sydney, 204 University of South Australia, 77 Monash University, 77	The University of Sydney, 38 La Trobe University, 32 Queensland University of Technology, 28	University of Newcastle, 85 University of Sydney, 51 James Cook University, 42	University of South Australia, 85 Monash University, 56 La Trobe University, 52	University of South Australia, 26 University of Sydney, 23 La Trobe, 21 Queensland University of Technology, 21	La Trobe University, 157 University of Melbourne, 116 University of Queensland, 37	La Trobe University, 102 University of NSW, 23 University of Wollongong, 21	James Cook University, 44 University of Newcastle, 27 University of Western Australia, 18

**TABLE 3:** Most globally cited articles.

Research stream	Authors (year)	Publication title	Journal	Citations (*n*)	Average citations per year
Diabetes‐related foot disease	Bergin and Wraight (2006)	Silver‐based wound dressings and topical agents for treating diabetic foot ulcers	*Cochrane Database of Systematic Reviews*	151	8
	Delbridge (1988)	Limited joint mobility in the diabetic foot: relationship to neuropathic ulceration	*Diabetic Medicine*	144	4
	Tapp (2003)	Foot complications in type 2 diabetes: an Australian population‐based study	*Diabetic Medicine*	135	6
Musculoskeletal	Redmond (2006)	Development and validation of a novel rating system for scoring standing foot posture: The Foot Posture Index	*Clinical Biomechanics*	661	35
	McKay (2001)	Ankle injuries in basketball: Injury rate and risk factors	*British Journal of Sports Medicine*	500	21
	Hiller (2006)	The Cumberland Ankle Instability Tool: A Report of Validity and Reliability Testing	*Archives of Physical Medicine & Rehabilitation*	427	22
Paediatrics	Baker (2009)	The Gait Profile Score and Movement Analysis Profile	*Gait & Posture*	454	28
	Dodd (2003)	A randomised clinical trial of strength training in young people with cerebral palsy	*Developmental Medicine & Child Neurology*	233	10
	Blundell (2003)	Functional strength training in cerebral palsy: A pilot study of a group circuit training class for children aged 4–8 years	*Clinical Rehabilitation*	174	8
Surgery	Khan (1992)	Outcome of conservative and surgical management of navicular stress fracture in athletes: Eighty‐six cases proven with computerised tomography	*American Journal of Sports Medicine*	160	5
	Wilde (1994)	Resection for symptomatic talocalcaneal coalition	*Journal of Bone & Joint Surgery*	140	5
	Hutchins (1985)	Long‐term results of early surgical release in club feet	*Journal of Bone & Joint Surgery*	123	3
Neurovascular	Kerr (2010)	Predictors of future falls in Parkinson’s disease	*Neurology*	417	26
	Hillier (2015)	Assessing Proprioception: A Systematic Review of Possibilities	*Neurorehabilitation & Neural Repair*	209	19
	Brooks (2001)	TBI or not TBI: That is the question. Is it better to measure toe pressure than ankle pressure in diabetic patients?	*Diabetic Medicine*	190	8
Workforce	Chisholm (2011)	Measuring rural allied health workforce turnover and retention: What are the patterns, determinants and costs	*Australian Journal of Rural Health*	69	5
	Munro (1998)	Foot‐care awareness. A survey of persons aged 65 years and older	*Journal of the American Podiatric Medical Association*	59	2
	Lawrence (2021)	Weight bias among healthcare professionals: A systematic review and meta‐analysis	*Obesity*	49	10
Dermatology	McInnes (2008)	Support surfaces for pressure ulcer prevention	*Cochrane Database of Systematic Reviews*	235	14
	Lacouture (2013)	Analysis of dermatologic events in vemurafenib‐treated patients with melanoma	*Oncologist*	173	14
	Tan (2007)	Subungual melanoma: A study of 124 cases highlighting features of early lesions, potential pitfalls in diagnosis and guidelines for histologic reporting	*American Journal of Surgical Pathology*	146	8
Rheumatology	Hodge (1999)	Orthotic management of plantar pressure and pain in rheumatoid arthritis	*Clinical Biomechanics*	201	8
	Bennell (2011)	Lateral wedge insoles for medial knee osteoarthritis: 12‐month randomised controlled trial	*BMJ*	160	11
	Sturnieks (2004)	Physiological risk factors for falls in older people with lower limb arthritis	*Journal of Rheumatology*	152	7
Gerontology	Menz (2006)	Foot and ankle risk factors for falls in older people: A prospective study	*Journals of Gerontology Series A: Biological Sciences and Medical Sciences*	327	17
	Menz (2005)	Foot and ankle characteristics associated with impaired balance and functional ability in older people	*Journals of Gerontology Series A: Biological Sciences and Medical Sciences*	325	16
	Scott (2007)	Age‐related differences in foot structure and function	*Gait & Posture*	246	14
First Nations foot health	Jeyaraman (2019)	Mortality in patients with diabetic foot ulcer: A retrospective study of 513 cases from a single Centre in the Northern Territory of Australia	*BMC Endocrine Disorders*	79	13
	McDermott (2001)	Improving diabetes care in the primary healthcare setting: A randomised cluster trial in remote Indigenous communities	*Medical Journal of Australia*	73	3
	Bailie (2007)	Improving organisational systems for diabetes care in Australian Indigenous communities	*BMC Health Services Research*	55	3

Table 4 contains information relating to the NHMRC level of evidence, HERDC funding category and UKCRC Health Research Classification System category for all publications included in this review. The highest proportion of category 1 funding was reported in the *rheumatology* stream, with 47% (*n* = 42) of studies receiving this level of support. In contrast, the *diabetes‐related foot disease* stream had the lowest proportion, with only 3% (*n* = 25) of studies funded at category 1 level. Regarding evidence quality, the *musculoskeletal and sports* stream produced the greatest volume of level I evidence (*n* = 68, 22%), whereas the *surgery* stream had the lowest (*n* = 6, 3%).

**TABLE 4:** Characteristics of publications stratified by NHMRC level of evidence, HERDC funding category and UKCRC Health Research Classification System category.

	Diabetes related foot disease	Musculoskeletal	Paediatrics	Surgery	Neurovascular	Workforce	Dermatology	Rheumatology	Gerontology	First Nations foot health
Total number of publications, *n*	335	308	280	184	121	105	91	89	81	47
NHMRC level of evidence, *n (%)*										
1	10 (3)	68 (22)	33 (12)	6 (3)	23 (19)	5 (5)	11 (12)	11 (12)	6 (7)	5 (11)
2	35 (10)	80 (26)	19 (6)	18 (10)	7 (6)	5 (5)	19 (21)	15 (17)	6 (7)	1 (2)
3	47 (14)	92 (30)	181 (65)	47 (26)	25 (20)	10 (10)	26 (29)	51 (57)	22 (28)	13 (28)
4	243 (73)	68 (22)	47 (17)	113 (61)	66 (55)	85 (80)	35 (38)	12 (14)	47 (58)	28 (59)
HERDC funding category, *n (%)*										
1—Australian competitive grant	25 (7)	46 (15)	73 (26)	9 (5)	10 (8)	12 (11)	6 (7)	42 (47)	32 (40)	15 (33)
2—Other public sector	27 (8)	24 (8)	12 (4)	3 (1)	4 (3)	8 (8)	2 (2)	6 (7)	10 (12)	14 (30)
3—Industry and other	72 (22)	32 (10)	41 (15)	12 (6)	9 (8)	22 (20)	26 (29)	12 (13)	9 (11)	2 (4)
4—Cooperative research centre	2 (1)	0 (0)	0 (0)	0 (0)	3 (2)	7 (7)	3 (3)	0 (0)	0 (0)	0 (0)
0—No funding	207 (62)	206 (67)	154 (55)	160 (87)	95 (79)	56 (54)	54 (59)	29 (33)	30 (37)	15 (33)
UKCRC Health Research Classification System category, *n (%)*										
1—Underpinning research	0 (0)	1 (< 1)	13 (5)	9 (5)	0 (0)	41 (39)	0 (0)	0 (0)	1 (< 1)	1 (2)
2—Aetiology	116 (35)	101 (33)	65 (23)	28 (15)	21 (17)	1 (< 1)	46 (51)	26 (29)	48 (59)	1 (2)
3—Prevention of disease and conditions, and promotion of well‐being	3 (1)	1 (< 1)	1 (< 1)	5 (3)	0 (0)	1 (< 1)	3 (3)	1 (< 1)	1 (< 1)	1 (2)
4—Detection, screening and diagnosis	42 (12)	57 (18)	107 (38)	19 (10)	76 (63)	0 (0)	5 (5)	5 (6)	7 (9)	23 (49)
5—Development of treatments and therapeutic interventions	2 (1)	3 (< 1)	4 (1)	7 (4)	4 (3)	0 (0)	0 (0)	16 (18)	9 (11)	0 (0)
6—Evaluation of treatments and therapeutic interventions	78 (23)	117 (38)	79 (28)	94 (51)	20 (17)	2 (2)	26 (29)	35 (39)	14 (17)	4 (9)
7—Management of diseases and conditions	39 (12)	23 (7)	10 (4)	19 (10)	0 (0)	0 (0)	5 (5)	5 (6)	0 (0)	1 (2)
8—Health and social care services research	54 (16)	5 (2)	1 (< 1)	3 (2)	0 (0)	60 (57)	6 (7)	1 (< 1)	1 (< 1)	16 (34)

Abbreviations: HERDC, Higher Education Research Data Collection; NHMRC, National Health and Medical Research Council and UKCRC, United Kingdom Clinical Research Commission.

The following sections summarise the results of each of the 10 podiatry‐relevant research streams.

### 3.1 Dermatology

A total of 91 Australian publications related to dermatology were identified from 1978 to 2024. Of these, 11 (12%) provided level I evidence, 19 (21%) provided level II evidence, 26 (29%) provided level III evidence and 35 (38%) provided level IV evidence. The majority of publications (*n* = 54; 59%) did not receive research funding. The most common source of funding was from category 3 (industry) (*n* = 26; 29%), whereas the least common was from category 2 (other/public sector) (*n* = 2; 2%). The Australian dermatology publications focused most frequently on ‘aetiology’ (*n* = 46; 51%) with no research completed in development of treatment and therapeutic interventions or underpinning research.

### 3.2 Diabetes‐Related Foot Disease

A total of 335 Australian publications related to diabetes‐related foot disease were identified from 1983 to 2024. Of these, 10 (3%) provided level I evidence, 35 (10%) provided level II evidence, 47 (14%) provided level III evidence and 243 (73%) provided level IV evidence. The majority of publications (*n* = 207; 62%) did not receive research funding. Of the 126 (38%) publications that received funding, the most common source of funding was from category 3 (*n* = 72; 22%), whereas the least common was from category 4 (*n* = 2; < 1%). The Australian publications on diabetes‐related foot disease focused most frequently on ‘aetiology’ (*n* = 116; 35%) and least frequently on ‘development of treatments and therapeutic interventions’ (*n* = 2; < 1%).

### 3.3 First Nations Foot Health

A total of 47 Australian publications related to First Nations foot health were identified from 1998 to 2024. There were 5 (11%) publications with level I evidence, 1 (2%) with level II evidence, 13 (28%) with level III evidence and 28 (60%) with level IV evidence. Approximately one‐third of publications (*n* = 15; 32%) did not receive research funding. Of the 32 (68%) publications that received funding, most were supported by category 4 funding (*n* = 16; 34%). In contrast, category 1 funding (*n* = 15; 32%) was the least common funding source. The Australian publications related to First Nations foot health focused most frequently on ‘detection, screening, diagnosis’ (*n* = 23; 51%) and least frequently on ‘prevention of disease and conditions, and promotion of well‐being’ (*n* = 1; 2%).

### 3.4 Gerontology

A total of 81 Australian publications related to gerontology were identified from 1995 to 2024. Of these, 6 (7%) provided level I evidence, 6 (7%) provided level II evidence, 22 (27%) provided level III evidence and 47 (58%) provided level IV evidence. There were 30 (37%) publications that did not receive research funding. The most common source of funding reported for the remaining publications was from category 1 (*n* = 32; 40%), whereas the least commonly reported was from category 3 (*n* = 9; 11%). Most of the Australian gerontology publications focused on ‘aetiology’ (*n* = 48; 59%), whereas ‘prevention of disease and conditions, promotion of well‐being’ and ‘health and social care services research, and underpinning research’ (*n* = 1 per category; 1%) were the least focused on.

### 3.5 Musculoskeletal and Sports

A total of 308 Australian publications related to musculoskeletal and sports aspects of the foot and ankle were identified from 1991 to 2024. There were 68 (22%) publications with level I evidence, 80 (26%) with level II evidence, 92 (30%) with level III evidence and 68 (22%) with level IV evidence. A total of 206 (66.9%) publications reported no research funding. The most frequent funding source for the remaining publications was from category 1 (*n* = 46; 19.9%), whereas the least frequent was from category 2 (*n* = 24; 68%). The majority of Australian publications related to musculoskeletal aspects of the foot and ankle focused on ‘evaluation of treatments and therapeutic interventions’ (*n* = 117; 38%). In contrast, ‘underpinning research’ and ‘prevention of disease and conditions, and promotion of well‐being’ (*n* = 1 for each category; < 1%) were the least researched areas.

### 3.6 Neurological and Vascular

A total of 121 Australian publications related to neurological and vascular aspects of the foot and ankle were identified from 1981 to 2024. There were 23 (19%) publications with level I evidence, 7 (6%) with level II evidence, 25 (21%) with level III evidence and 66 (55%) with level IV evidence. A total of 95 (79%) publications did not receive research funding. For the remaining 26 (21%) publications, the most common source of funding was from category 1 (*n* = 10; 8%), whereas the least common was from category 4 (*n* = 3; 2%). The majority of Australian publications related to neurological and vascular aspects of the foot and ankle were focused on ‘detection, screening and diagnosis’ (*n* = 76; 63%). In contrast, ‘development of treatments and therapeutic interventions’ (*n* = 4; 3%) was the least focused area of research.

### 3.7 Paediatrics

A total of 280 Australian publications related to paediatrics were identified from 1980 to 2024. Of these, 33 (12%) provided level I evidence, 19 (7%) provided level II evidence, 181 (65%) provided level III evidence and 47 (17%) provided level IV evidence. A total of 259 (89%) publications did not receive research funding. The most common source of funding for the remaining publications was from category 1 (*n* = 70; 25%), whereas the least common was from category 2 (*n* = 15; 5%). The Australian publications on paediatrics focused most frequently on ‘detection, screening and diagnosis’ (*n* = 107; 38%) and least frequently on ‘prevention of disease and conditions, and promotion of well‐being’ and ‘health and social care services research’ (*n* = 1; < 1%).

### 3.8 Rheumatology

A total of 89 Australian publications related to rheumatology were identified from 1983 to 2024. Of these, 11 (12%) provided level I evidence, 15 (17%) provided level II evidence, 51 (57%) provided level III evidence and 12 (14%) provided level IV studies. There were 29 (33%) publications with no research funding. For the remaining publications, the most common funding source was from category 1 (*n* = 42; 47%), whereas the least common was from category 2 (*n* = 6; 7%). The Australian rheumatology publications focused most frequently on ‘evaluation of treatments and therapeutic interventions’ (*n* = 35; 39%) and least frequently on ‘prevention of diseases and conditions, and promotion of well‐being’ and health and social care services research (*n* = 1; 1%).

### 3.9 Workforce and Education

A total of 105 Australian workforce and education publications were identified from 1998 to 2024. Of these, 5 (5%) provided level I evidence, 5 (5%) provided level II evidence, 10 (9%) provided level III evidence and 85 (81%) provided level IV evidence. A total of 56 (53%) publications did not receive research funding. Of the publications that were funded, the most common funding source was from category 3 (*n* = 22; 21%), whereas the least common was from category 4 (*n* = 7; 7%). The Australian workforce and education publications focused most frequently on ‘health and social care services research’ (*n* = 60; 57%) and least frequently on ‘aetiology’ and ‘prevention of disease and conditions, and promotion of well‐being’ (*n* = 1; 1%).

### 3.10 Surgery

A total of 184 Australian publications related to foot and ankle surgery were identified from 1980 to 2024. Of these, 6 (3%) provided level I evidence, 18 (10%) provided level II evidence, 47 (25%) provided level III evidence and 113 (61%) provided level IV evidence. A total of 160 (87%) publications did not receive research funding. Of the remaining publications, the most common source of funding was from category 3 (*n* = 12; 6%), whereas the least common was from category 2 (*n* = 3; 2%). The Australian publications relating to foot and ankle surgery focused most frequently on the ‘evaluation of treatments and therapeutic interventions’ (*n* = 94; 51%) and least frequently on ‘health and social care services research’ (*n* = 3; 2%).

## 4 Discussion

This comprehensive bibliometric analysis represents the first mapping of Australian podiatry‐relevant research across multiple clinical areas: dermatology, diabetes‐related foot disease, First Nations foot health, gerontology, musculoskeletal and sports, neurological and vascular, paediatrics, rheumatology, foot and ankle surgery and workforce and education. The findings reveal a diverse research landscape, characterised by substantial growth in publication volume over time, particularly in the past 20 years. However, there were substantial variations in funding support, level of evidence generated and categories of research.

The overall publication volume (*n* = 1639) demonstrates evolution of podiatry and lower limb health research in Australia, with diabetes‐related foot disease (*n* = 335) and musculoskeletal and sports research (*n* = 308) emerging as the most productive areas. This distribution reflects both the perceived clinical burden of some conditions over others in podiatry practice, and the subsequent research culture that potentially has developed as a result. However, the substantial variation in publication volume across streams highlights disparities in research attention that may not align with clinical need or population health priorities. The level of evidence generated across streams reveals critical gaps in high‐quality research. Although the *musculoskeletal and sports* stream had the highest proportion of level 1 evidence (22%), most streams remained heavily weighted towards lower levels of evidence, with diabetes‐related foot disease showing the greatest proportion of level IV evidence (72%). This suggests that although Australian podiatry research has achieved substantial volume, it may not be of sufficient impact to drive evidence‐based practice change in some key clinical areas.

Across all streams, the majority of research was conducted without reported funding support, with the foot and ankle surgery stream reporting the highest proportion of nonfunded research (87%). This finding is consistent with previous bibliometric analyses [10, 11], which demonstrates the resilience of Australian researchers in producing outputs despite a lack of available funding. However, the relationship between funding availability and the quality of evidence is particularly evident when comparing streams, such as rheumatology, which achieved the highest level of competitive funding (47%, Category 1) and correspondingly higher proportion of level II evidence, with surgical research, which had minimal funding and predominantly level IV evidence. This suggests that more funding should be allocated to areas where higher levels of evidence, and the consequential evidence‐based practice guidance, is required.

The research categorisation using the UKCRC Health Research Classification system demonstrated important patterns in the types of Australian podiatry research that have been conducted to date. Across the different streams, ‘aetiology’ and ‘evaluation of treatment’ and ‘therapeutic interventions’ were most frequent, with ‘prevention of disease and conditions’ and ‘development of treatment and therapeutic interventions’ substantially underrepresented. This distribution suggests that Australian podiatry research has focused primarily on understanding existing issues and evaluating current treatment rather than developing innovative interventions or prevention strategies. This pattern of research is also consistent with research completed with little funding, where resource limitations compel researchers toward more feasible observational and evaluation studies rather than resource intensive prevention trials, clinical trials or intervention development.

The citation patterns observed across streams provide insight into research impact and influence. Gerontology research achieved the highest mean citations per article (62.8), despite representing one of the smaller publication volumes, suggesting that research in this area addresses fundamental questions with broad clinical relevance. Conversely, workforce and education research, although addressing critical professional development needs, achieved the lowest citation rates (11.9), potentially reflecting the more localised applicability of these findings or publication in speciality journals with smaller reach.

The funding disparities and dominance of level IV evidence across most streams highlight a critical challenge for the advancement of podiatry research in Australia. Strategic investment through competitive funding programmes could substantially improve the quality and impact of Australian podiatry research across all clinical streams, and likely result in an increase in the level of generated evidence.

### 4.1 Limitations

The findings of this study should be considered in light of several limitations. Firstly, as Scopus is a curated database that focuses on peer‐reviewed scientific content from journals, conference proceedings and books [12], so most grey literature is excluded as it typically does not undergo formal peer review or is not published in an indexed serial. Secondly, First Nations research is frequently published in nontraditional academic journals, and therefore, many important articles may have been excluded from the analysis. Furthermore, exploration of quality and rigour within this stream should be against First Nations directed parameters [13]. Thirdly, the restriction of musculoskeletal and sports to the foot and ankle may limit the comprehensiveness of our findings to the full scope of the broader Australian podiatry research landscape. The exclusion of lab‐based studies may have also disproportionately reduced the scope of research in specific streams, such as biomechanical lab‐based studies in the musculoskeletal and sports stream. Alas, owing to the interdisciplinary nature of many publications, categorising certain articles into a single research specialisation proved challenging as their content spanned multiple domains. Fourth, in this review, 164 articles (10%) were classified under more than one research specialisation. As manual coding of research categorisation, level of evidence and funding was completed by a number of different researchers, some papers may have been misclassified. Two researchers (P.T. and M.C.) cleaned and checked the data to reduce the likelihood of misclassification. Under‐reporting of funding sources is possible and may not accurately reflect actual financial research support. Finally, citation metrics are influenced by citation age and may not fully represent the clinical relevance of practice impact.

## 5 Conclusion

Australian podiatry research has grown substantially over the past 2 decades. However, significant disparities exist in research volume, evidence quality and funding support across different streams. Although diabetes‐related foot disease and musculoskeletal and sports research achieved the highest publication volumes, most streams remained dominated by level IV evidence. This could be a result of the majority of research conducted and published not reporting external funding, which is a critical barrier to creating higher levels of evidence to influence practice change. Rheumatology research, which secured the highest proportion of competitive funding, correspondingly produced higher levels of evidence. This provides evidence that strategic investment is required to improve research quality across all streams. The predominance of aetiological and treatment evaluation studies, with limited prevention and intervention development research, may not optimally address clinical needs. These findings will inform national research priority‐setting and highlight the need for strategic investment to enhance the quality and clinical relevance of Australian podiatry research.

## Author Contributions

**Peta Tehan:** conceptualisation, data curation, formal analysis, writing – original draft. **Ameer Nor Azhar:** data curation, writing – review and editing. **Helen Banwell:** data curation, writing – review and editing. **Shan Bergin:** data curation, writing – review and editing. **James Charles:** data curation, writing – review and editing. **Fiona Hawke:** data curation, writing – review and editing. **Malia Ho:** data curation, writing – review and editing. **Sheree Hurn:** data curation, writing – review and editing. **Michelle Kaminski:** data curation, writing – review and editing. **Polly Lim:** data curation, writing – review and editing. **Saraid Martin:** data curation, writing – review and editing. **Hylton B. Menz:** data curation, writing – review and editing. **John Osborne:** data curation, writing – review and editing. **Benjamin Peterson:** data curation, writing – review and editing. **Dean Samaras:** data curation, writing – review and editing. **Cylie Williams:** data curation, writing – review and editing. **Matthew R. Carroll:** data curation, formal analysis, writing – review and editing.

## Conflicts of Interest

The authors declare no conflicts of interest.

## Supporting information


Supporting Information S1

## Data Availability

Data are available upon reasonable request.
