# Supplementary material for: An Overview of Australian Podiatry Research: A Bibliometric Review
Source: J Foot Ankle Res. 2026 Feb 14;19(1):e70113. doi: 10.1002/jfa2.70113 (PMC12906308; doi:10.1002/jfa2.70113)
Supplement: Supplementary file 1 — Supporting Information S1 [file JFA2-19-e70113-s001.docx]

Supplementary Table 1 : Evidence Hierarchy Table

| **Level** | **Intervention** | **Diagnosis** | **Prognosis** | **Aetiology** | **Screening** |
| --- | --- | --- | --- | --- | --- |
| **I** | A systematic review of Level II studies | A systematic review of Level II studies | A systematic review of Level II studies | A systematic review of Level II studies | A systematic review of Level II studies |
| **II** | A randomised controlled trial | A study of test accuracy with an independent, blinded comparison with a valid reference standard, among consecutive patients with a defined clinical presentation | A prospective cohort study | A prospective cohort study | A randomised controlled trial |
| **III-1** | A pseudorandomised controlled trial (i.e. alternate allocation of some other method) | A study of test accuracy with an independent, blinded comparison with a valid reference standard, among consecutive patients with a defined clinical presentation | All or none | All or none | A pseudorandomised controlled trial (i.e. alternate allocation of some other method) |
| **III-2** | A comparative study with concurrent controls:  • Non-randomised, experimental trial  • Cohort study  • Case-control study  • Interrupted time series with a control group | A comparison with reference standard that does not meet the criteria required for Level II and III-1 | Analysis of prognostic factors amongst untreated control patients in a randomised controlled trial | A retrospective cohort study | A comparative study with concurrent controls:  • Non-randomised, experimental trial  • Cohort study  • Case-control study |
| **III-3** | A comparative study without concurrent controls:  • Historical control study  • Two or more single arm study  • Interrupted time series without a parallel control group | Diagnostic case-control study | A retrospective cohort study | A case-control study | A comparative study without concurrent controls:  • Historical control study  • Two or more single arm study |
| **IV** | Case studies with either post-test or pre-test/post-test outcomes | Study of diagnostic yield (no reference standard) | Case series, or cohort study of patients at different stages of disease | A cross-sectional study | Case studies |

Supplementary Table 2: UKCRC Health Research Classification System

| **Research Activity** | **Description** |
| --- | --- |
| **1. Underpinning Research** | Research that underpins investigations into the cause, development, detection, treatment and management of diseases, conditions and ill health |
| **2. Aetiology** | Identification of determinants that are involved in the cause, risk or development of disease, conditions and ill health |
| **3. Prevention of Disease and Conditions, and Promotion of Well-being** | Research aimed at the primary prevention of disease, conditions or ill health, or promotion of well-being |
| **4. Detection, Screening and Diagnosis** | Discovery, development and evaluation of diagnostic, prognostic and predictive markers and technologies |
| **5. Development of Treatments and Therapeutic Interventions** | Discovery and development of therapeutic interventions and testing in model systems and preclinical settings |
| **6. Evaluation of Treatments and Therapeutic Interventions** | Testing and evaluation of therapeutic interventions in clinical, community or applied settings |
| **7. Management of Diseases and Conditions** | Research into individual care needs and management of disease, conditions or ill health |
| **8. Health and Social Care Services Research** | Research into the provision and delivery of health and social care services, health policy and studies of research design, measurements and methodologies |
